# Aging impairs osteoblast differentiation of mesenchymal stem cells grown on titanium by favoring adipogenesis

**DOI:** 10.1590/1678-775720160037

**Published:** 2016

**Authors:** Rodrigo Paolo Flores ABUNA, Camila Tami STRINGHETTA-GARCIA, Leonardo Pimentel FIORI, Rita Cassia Menegati DORNELLES, Adalberto Luiz ROSA, Marcio Mateus BELOTI

**Affiliations:** 1 - Universidade de São Paulo, Faculdade de Odontologia de Ribeirão Preto, Laboratório de Cultura de Células, Ribeirão Preto, SP, Brasil.; 2 - Universidade Estadual Paulista, Faculdade de Odontologia de Araçatuba, Departamento de Ciências Básicas, Laboratório de Fisiologia Endócrina e do Envelhecimento, Araçatuba, SP, Brasil.

**Keywords:** Aging, Osteoblasts, Adipocytes, Stem cells, Dental implants, Titanium

## Abstract

Aging negatively affects bone/titanium implant interactions. Our hypothesis is that the unbalance between osteogenesis and adipogenesis induced by aging may be involved in this phenomenon.

**Objective:**

We investigated the osteoblast and adipocyte differentiation of mesenchymal stem cells (MSCs) from young and aged rats cultured on Ti.

**Material and Methods:**

Bone marrow MSCs derived from 1-month and 21-month rats were cultured on Ti discs under osteogenic conditions for periods of up to 21 days and osteoblast and adipocyte markers were evaluated.

**Results:**

Cell proliferation, alkaline phosphatase (ALP) activity, extracellular matrix mineralization and gene expression of RUNX2, osterix, ALP, bone sialoprotein, osteopontin, and osteocalcin were reduced in cultures of 21-month rats compared with 1-month rats grown on Ti. Gene expression of PPAR-γ , adipocyte protein 2, and resistin and lipid accumulation were increased in cultures of 21-month rats compared with 1-month rats grown on the same conditions.

**Conclusions:**

These results indicate that the lower osteogenic potential of MSCs derived from aged rats compared with young rats goes along with the higher adipogenic potential in cultures grown on Ti surface. This unbalance between osteoblast and adipocyte differentiation should be considered in dental implant therapy to the elderly population.

## INTRODUCTION

Titanium (Ti) implants have been largely used in dentistry thanks to their physical properties and superior ability to osseointegrate^2^. In addition to the implant characteristics, the clinical success of this treatment depends on several aspects such as surgical procedure, patient health conditions, and local bone quality and quantity^4,10,21^.

The implantology research has been focused on the development of surface modifications that result in unique topography and chemical features that may regulate cell adhesion, proliferation and differentiation, and ultimately the interfacial tissue formation^5,6,9,17,18,20^. Regarding the quality and amount of bone tissue, they are affected by various factors such as heart/vascular disease, estrogen deficiency, diabetes, and aging^12,15,19^. Aging also increases the risk of bone fracture and impairs the healing process in humans and animal models^8,13,22^. It has been shown that the delay in fracture repair and the reduced bone volume in the healing site in aged mice is associated with a decreased proliferation of stem cells and a disrupted osteoblast differentiation^13,22^.

The effects of aging on bone repair indicate that the process of Ti implant osseointegration could also be regulated by the age of the patient. Indeed, the number of mineralized nodules and the calcium content were reduced in osteoblasts derived from old patients compared with young patients when cultured on Ti surface^23^. Corroborating this finding, the alkaline phosphatase (ALP) activity and osteocalcin (OC) expression were downregulated in osteoblasts derived from aged rats compared with young ones when they were grown on Ti surface with different surface topographies^16^. Furthermore, a discrete but significant decrease in bone-to-implant contact was observed in one of the Ti surfaces placed into mouse femurs^16^.

Despite the clear evidences of the negative effects of aging on osteoblast and bone tissue interactions with Ti implants, the mechanisms behind this phenomenon have not been fully understood yet. Then, we hypothesized that the unbalance between osteogenesis and adipogenesis induced by aging may be one of the factors involved in the reduced osteoblast response to Ti surfaces. Thus, in the present study, we investigated the osteoblast and adipocyte differentiation of mesenchymal stem cells (MSCs) derived from bone marrow of young and aged rats cultured on Ti surface.

## MATERIAL AND METHODS

### Preparation of Ti discs

Discs of commercially pure grade 2 Ti (Realum, São Paulo, SP, Brazil) with 12 mm in diameter and 1.5 mm thick were polished using 320 and 600 grit silicon carbide, cleaned by sonication and rinsed with toluene and deionized H_2_O several times, autoclaved and air-dried as previously described^18^.

### Cell culture

The Committee of Ethics in Animal Research approved all animal procedures performed during the experiments (Approval number: 11.1.890.53.0). Bone marrow MSCs were obtained from the tibiae of 1-month and 21-month female Wistar rats and cultured in growth medium consisting of α-MEM (Invitrogen-Gibco, Grand Island, NY, USA) supplemented with 15% fetal bovine serum (Gibco), 50 µg/mL gentamycin (Gibco), 50 µg/mL vancomycin (Acros Organics, Geel, Belgium), and 0.3 µg/mL fungizone (Gibco) until reaching subconfluence. Then, MSCs were cultured in osteogenic medium containing growth medium plus 5 µg/ml ascorbic acid (Gibco), 7 mM β-glycerophosphate (Sigma-Aldrich, St. Louis, MO, USA), and 10^-7^ M dexamethasone (Sigma) on Ti discs for periods of up to 21 days. During the culture period, cells were incubated at 37°C in a humidified atmosphere of 5% CO_2_, and the medium was changed every three days.

### Cell proliferation

Cell proliferation was evaluated at days 4, 7, and 10 with 3-(4,5-dimethylthiazol-2-yl)-2,5-diphenyltetrazolium bromide (MTT, Sigma-Aldrich). Cultures were incubated with 2 mL of MTT (5 mg/ml) in phosphate-buffered saline at 37°C. After 4 h, the solution was removed and 1 mL of acid isopropanol (0.04 N HCl in isopropanol) was added. After shaking for 5 min, 150 µL of this solution was used to read the optical density at 570 nm (µQuant, Bio-Tek, Winooski, VT, USA). Data were obtained in quintuplicate (n=5) and expressed as absorbance.

### Alkaline phosphatase (ALP) activity

Alkaline phosphatase activity was determined at days 4, 7, and 10 by measuring the release of thymolphthalein from thymolphthalein monophosphate using a commercial kit (Labtest Diagnostica SA, Lagoa Santa, MG, Brazil). Briefly, 50 μL of thymolphthalein monophosphate was mixed with 0.5 mL of 0.3 M diethanolamine buffer, pH 10.1, and kept for 2 min at 37°C before the addition of 50 μL of cell lysates obtained by five cycles of thermal shock (-20°C for 20 min and 37°C for 15 min). Then, after 10 min at 37°C, the reaction was stopped by adding 2 mL of Na_2_CO_3_ (0.09 mmol/mL) and NaOH (0.25 mmol/mL) solution, and the optical density was measured at 590 nm (µQuant, Bio-Tek). Data were obtained in quintuplicate (n=5) and expressed as ALP activity normalized by total protein content, which was determined by the Lowry method^14^.

### Extracellular matrix mineralization

Extracellular matrix mineralization was detected at day 21 by alizarin red staining (Sigma-Aldrich). Cells were fixed in 10% formalin for 2 h at room temperature, dehydrated and stained with 2% alizarin red pH 4.2 (Sigma-Aldrich) for 10 min. For qualitative analysis, culture images were captured with a high-resolution digital camera (Canon EOS Digital Rebel Camera, Canon, Lake Success, NY, USA). Then, calcium content was evaluated using a colorimetric method. Briefly, 280 μL of 10% acetic acid was added to each well stained with alizarin red, and the plate was incubated at room temperature for 30 min under shaking. The slurry was overlaid with 100 μL of mineral oil (Sigma-Aldrich), heated to 85°C for 10 min and transferred to ice for 5 min. The slurry was then centrifuged at 20,000 g for 15 min, and 100 μL of supernatant was transferred to a microcentrifuge tube with 40 μL of 10% ammonium hydroxide to neutralize the acid. The optical density was read at 405 nm (µQuant, Bio-Tek), and data were obtained in quintuplicate (n=5) and expressed as absorbance.

### Gene expression

Quantitative real-time polymerase chain reaction (PCR) was performed on day 10 to evaluate the gene expression of runt-related transcription factor 2 (RUNX2), osterix (OSX), ALP, bone sialoprotein (BSP), OC, osteopontin (OPN), peroxisome proliferator-activated receptor (PPAR-γ), adipocyte protein 2 (aP2), and resistin (RTN). Total RNA was extracted with Trizol reagent (Life-Technologies, Grand Island, NY, USA), and the concentration was determined by reading the optical density at the following different wavelengths: 260, 280, 230, and 320 nm (GE Healthcare, Milwaukee, WI, USA). Complementary DNA (cDNA) was synthesized using 1 µg of RNA through a reverse transcription reaction (Life Technologies-Applied Biosystems, Warrington, UK) according to the manufacturer’s instructions. Real-time PCR was performed in a CFX96 Real-Time PCR Detection System (Bio-Rad Laboratories, Philadelphia, PA, USA) using TaqMan (Applied Biosystems) probes for the target genes. The standard PCR conditions were 50°C (2 min), 95°C (10 min), 40 cycles of 95°C (15 s), and 60°C (1 min). The relative gene expression was calculated in relation to glyceraldehyde-3-phosphate dehydrogenase (GAPDH) expression and its respective control using the cycle threshold (Ct) method. This assay was performed in quadruplicate (n=4).

### Lipid accumulation

Lipid accumulation was detected at day 21 by oil red O (Sigma-Aldrich). Cells were fixed in 10% formalin for 2 h at room temperature, washed with isopropanol 60% (Merck-Germany) and stained with 0.3% oil red O (Sigma-Aldrich) for 10 min. For qualitative analysis, culture images were captured with a high-resolution digital camera (Canon EOS Digital Rebel Camera, Canon) and the lipid accumulation was measured using a colorimetric assay. The incorporated oil red O (Sigma-Aldrich) was extracted by incubation with 100% isopropanol for 10 min under shaking at room temperature. After appropriate dilution, this solution was read at 500 nm in a plate reader μQuant (BioTek), and the data were obtained in quintuplicate (n=5) and expressed as absorbance.

### Statistical analyses

The results are expressed as the mean±standard deviation and data obtained in three time-points were analyzed using two-way ANOVA followed by Student Newman Keuls *post hoc* test. Data obtained in one time-point were analyzed using Student’s t-test. For all experiments the level of signiﬁcance was set at p≤0.05.

## RESULTS

### Cell proliferation

**Figure 1:**
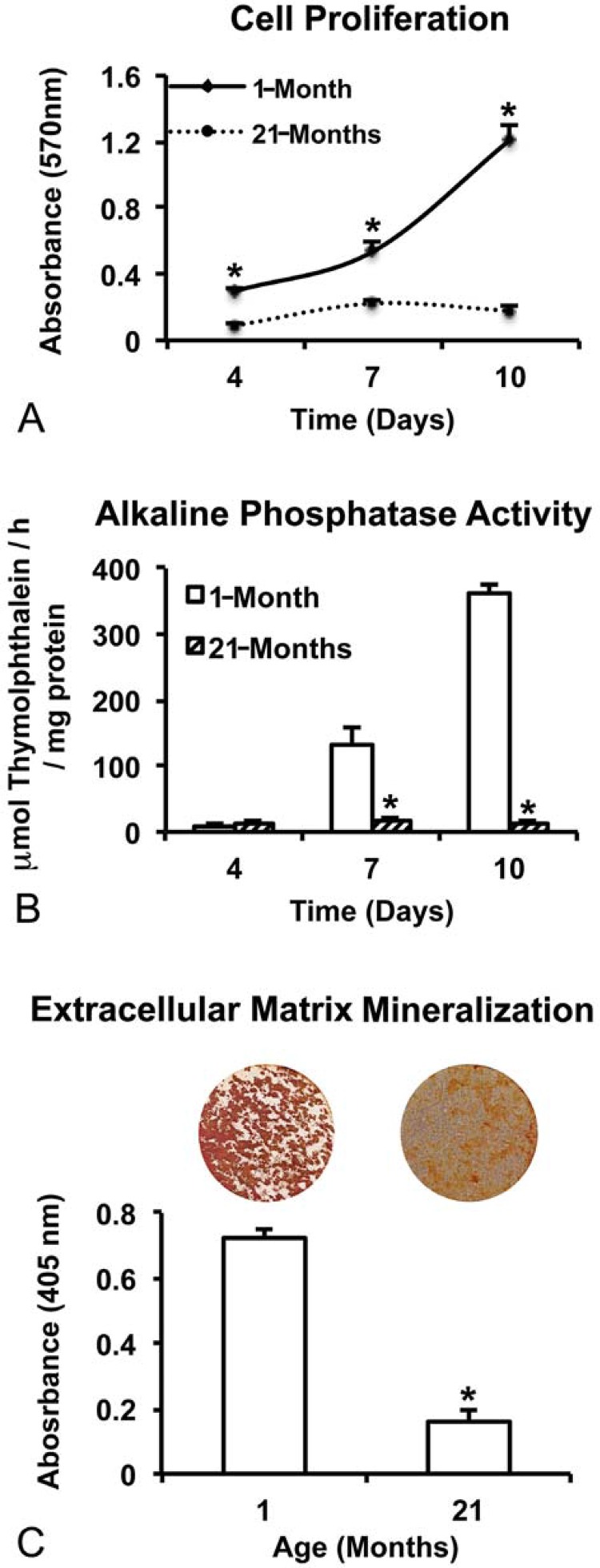
Proliferation (A) and alkaline phosphatase (ALP) activity at days 4, 7, and 10 (B) and extracellular matrix mineralization at day 21 (C) of cultures from 1-month and 21-month rats grown on Ti discs. Data are presented as the mean±standard deviation (n=5). * indicates significant differences between cells from 1-month and 21-month rats within each time point (p≤0.05)

Cell proliferation was higher in cultures from 1-month rats compared with 21-month rats at all evaluated time-points (p=0.001 for all periods; Figure 1A). Moreover, the cell number increased (p=0.001) over time in cultures from 1-month rats, while no significant difference (p=0.127) was observed between 7 and 10 days in cultures from 21-month rats (Figure 1A).

### ALP activity

Cells from 1-month rats showed higher ALP activity compared with cells from 21-month rats at 7 and 10 days (p=0.001 for both time-points), while no significant difference (p=0.561) was observed at 4 days (Figure 1B). In addition, the ALP activity increased (p=0.001) over time in cultures from 1-month rats, while no significant difference (p>0.151) was observed between 4, 7, and 10 days in cultures from 21-month rats (Figure 1B).

### Extracellular matrix mineralization

Extracellular calcium deposits were detected on Ti discs irrespective of cell source, with cells from 1-month rats producing the most dense and regularly distributed matrix (Figure 1C). Additionally, the calcium content was nearly five times higher (p=0.001) in cultures from 1-month rats compared with 21-month rats (Figure 1C).

### Gene expression

**Figure 2:**
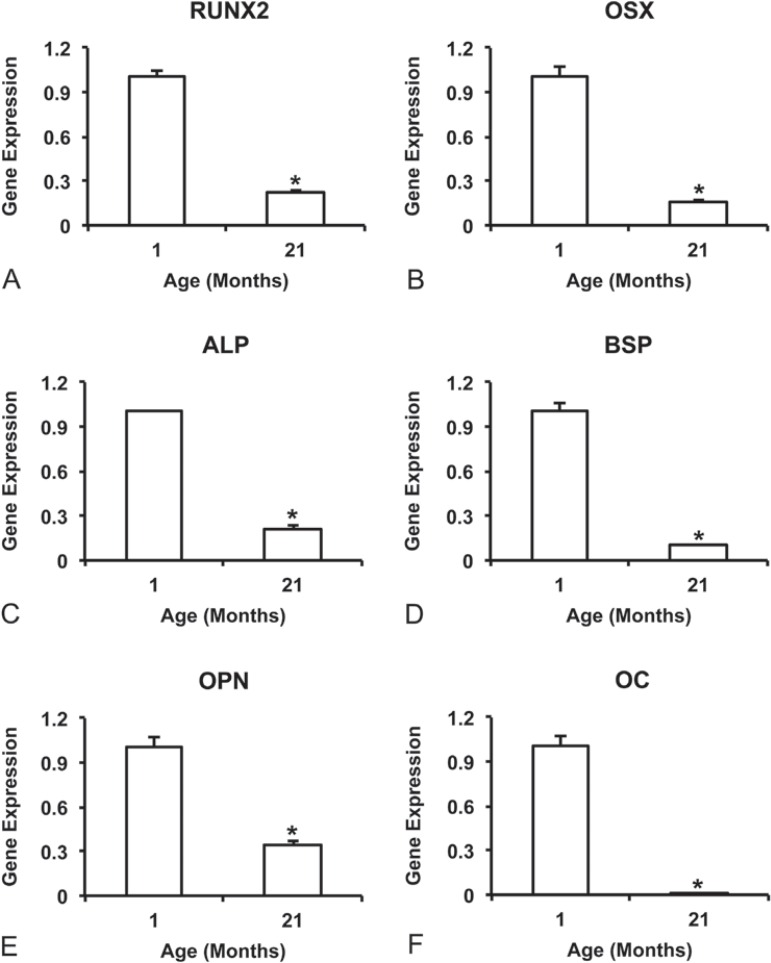
Gene expression at day 10 of the bone markers, RUNX2 (A), OSX (B), ALP (C), BSP (D), OPN (E), and OC (F) of cells from 1-month and 21-month rats grown on Ti discs. Data are presented as the mean±standard deviation (n=4). * indicates statistically significant differences between cells from 1-month and 21-month rats (p≤0.05)

**Figure 3:**
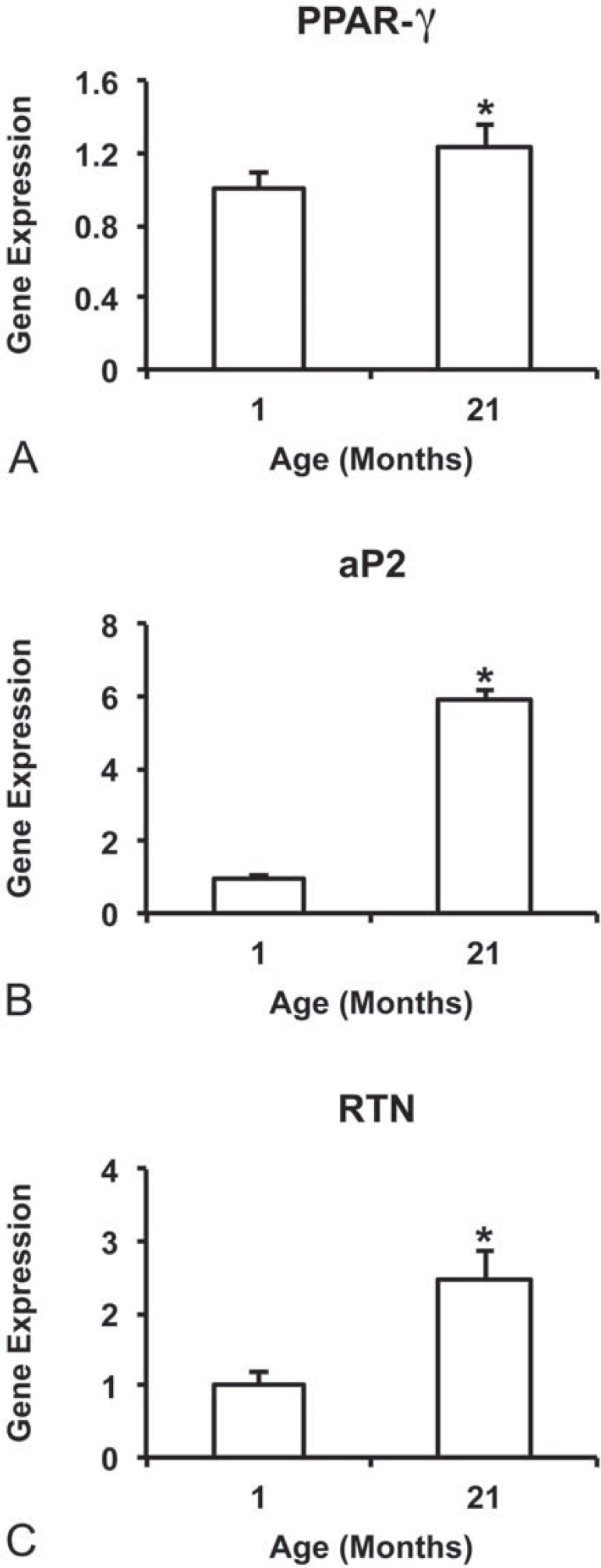
Gene expression at day 10 of the adipose tissue markers, PPAR-γ (A), aP2 (B), and RTN (C) of cells from 1-month and 21-month rats grown on Ti discs. Data are presented as the mean±standard deviation (n=4). * indicates statistically significant differences between cells from 1-month and 21-month rats (p≤0.05)

The gene expression of all evaluated bone markers, RUNX2 (Figure 2A), OSX (Figure 2B), ALP (Figure 2C), BSP (Figure 2D), OPN (Figure 2E), and OC (Figure 2F) was higher (p=0.001 for all genes) in cultures from 1-month rats compared with 21-month rats. On the other hand, the gene expression of all evaluated adipose tissue markers, PPAR-γ (p=0.007; Figure 3A), aP2 (p=0.050; Figure 3B), and RTN (p=0.001; Figure 3C) was higher in cultures from 21-month rats compared with 1-month rats.

### Lipid accumulation

**Figure 4:**
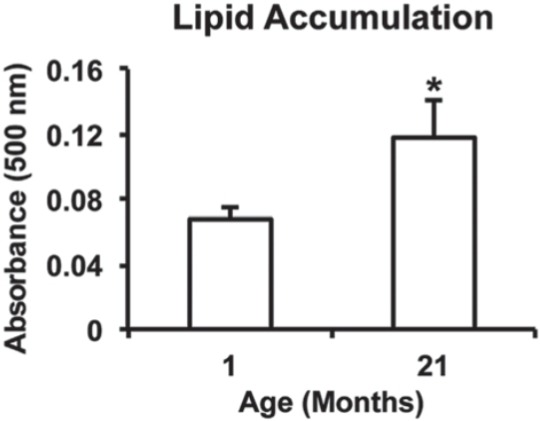
Lipid accumulation at day 21 of cultures from 1-month and 21-month rats grown on Ti discs. Data are presented as the mean±standard deviation (n=5). * indicates statistically significant differences between cells from 1-month and 21-month rats (p≤0.05)

Lipid accumulation was detected on Ti discs irrespective of cell source, with cells from 21-month rats producing the most dense lipid droplets (Figure 3). Additionally, the lipid content was nearly twice higher (p=0.010) in cultures from 21-month rats compared with 1-month rats (Figure 4).

## DISCUSSION

The present study was designed to investigate if disruption of the balance between osteogenesis and adipogenesis induced by aging may be one of the factors involved in the reduced osteoblast response to Ti surfaces. Our results showed that MSCs derived from 21-month rats exhibit lower osteogenic and higher adipogenic potential compared with MSCs derived from 1-month rats when cultured on Ti surface under osteogenic conditions.

The capacity to expand is a key feature of the osteoblastic cells in order to colonize the implant surface, and distinct results are reported regarding the effect of aging on cell proliferation^3^. Here, we observed a relevant decrease in the proliferation activity of cells from 21-month rats compared with 1-month rats cultured on Ti surface at all evaluated time-points. According to this result, a reduced cell number in osteoblast cultures derived from the calvaria of old rats compared with young ones was observed^16^. In contrast, MSCs from young and aged rats exhibited similar proliferation rate when cultured on polystyrene in a non-inducing differentiation medium^7^. Such discrepancies may be related to the use of cells in different stages of differentiation and culture conditions, since the studies were conducted with either osteoblastic cells in osteogenic medium or MSCs in growth medium.

Another relevant characteristic of osteoblastic cells to interact with implants is the ability to complete the differention process and to synthesize an extracellular matrix that will be subsenquently mineralized. In this study, all evaluated markers of osteoblast genotype and phenotype expression were higher in cultures derived from 1-month rats compared with 21-month rats, including gene expression of RUNX2, OSX, ALP, BSP, OPN and OC, ALP activity and extracellular matrix mineralization. Corroborating our findings, a reduced number of mineralized nodules and calcium content were noticed in osteoblasts derived from old patients compared with young ones grown on Ti surface^23^. Furthermore, a low osteogenic potential induced by aging was demonstrated *in vivo* in mice and *in vitro* in cells from rats in response to different Ti surfaces^16^.

The reduced osteoblast differentiation of MSCs grown on Ti surface induced by aging was paralleled by an increased genotype and phenotype expression level of adipocyte markers, such as higher gene expression of PPAR-γ, aP2, and RTN, and more lipid accumulation, the later not reported in studies investigating the effect of aging on the interaction between osteoblasts and Ti^16,23^. In contrast with this finding, it was reported that there were no differences between MSCs from young and aged rats in terms of osteoblast and adipocyte differentiation; however, this study evaluated cells obtained from 3-week and 12-month rats cultured on polystyrene under osteogenic and adipogenic conditions, respectively^7^. It is worth noting that, in our study, cells were cultured only in osteogenic medium on Ti surface, indicating that even in a non-adipogenic milieu aging may disrupt the balance between osteoblast and adipocyte differentiation in favor of adipogenesis. Also, it has been shown that adipocytes inhibit the osteoblast phenotype expression by releasing tumor necrosis factor alpha to the tissue environment^1^. These findings are of relevance from the clinical perspective as the process of Ti implant osseointegration occurs in a more osteogenic environment than in an adipogenic one.

The unbalance between osteoblast and adipocyte differentiation induced by aging could be related to the extracellular signal-regulated kinase 1/2 (ERK 1/2) that acts as a switch for the reciprocal regulation of osteogenesis and adipogenesis by modulating RUNX2 and PPAR-γ expression and/or activity^11^. While ERK 1/2 phosphorylation is sustained during osteoblast differentiation, it is transient and decreased during adipocyte differentiation of human MSCs, suggesting that a reduced ERK 1/2 activation favors adipogenesis^11^. However, further studies are necessary to investigate the participation of ERK 1/2 on the reduced osteoblast differentiation of MSCs cultured on Ti surface induced by aging.

## CONCLUSIONS

The results of our study indicate that the reduced osteogenic potential of MSCs derived from aged rats compared with young ones goes along with the increased adipogenic potential in cultures grown on Ti surface. In this context, the unbalance between osteoblast and adipocyte differentiation should be taken into consideration in dental implant therapy to the older-adult population.
